# Diminishing Efficacy of Second-Line Levofloxacin-Based Quadruple Therapy in *Helicobacter pylori* Eradication: A Prospective Real-World Study in Vietnam Amid Rising Antibiotic Resistance

**DOI:** 10.3390/antibiotics14080826

**Published:** 2025-08-14

**Authors:** Thong Duy Vo, Thao Thu Ngan, Thuy Thi Thanh Trinh

**Affiliations:** 1 Department of Internal Medicine, School of Medicine, University of Medicine and Pharmacy at Ho Chi Minh City, Ho Chi Minh City 71724, Vietnam; 2 Department of Gastroenterology, University Medical Center Ho Chi Minh City, 215 Hong Bang, Cho Lon Ward, Ho Chi Minh City 71724, Vietnam; 3 Faculty of Medicine, Nguyen Tat Thanh University, Ho Chi Minh City 71724, Vietnam

**Keywords:** *Helicobacter pylori*, levofloxacin, eradication therapy, second-line treatment, antibiotic resistance, bismuth, PALB

## Abstract

**Background/Objectives:** *Helicobacter pylori* (*H. pylori*) infection remains highly prevalent in Vietnam, associated with chronic gastritis, peptic ulcers, and gastric cancer. This study aimed to evaluate the real-world eradication rate of levofloxacin-based bismuth quadruple therapy (PALB) as second-line treatment, assess adherence, and identify associated factors with treatment success. **Methods:** We conducted a prospective cohort study including 225 patients with confirmed failure of classical bismuth-based quadruple therapy. All received a 14-day PALB regimen. *H. pylori* eradication was assessed using 13C-urea breath test and/or rapid urease test 4–12 weeks after treatment. **Results:** Eradication rates were 78.2% (mITT) and 78.6% (PP), with 95% CIs overlapping the 80% benchmark. Adherence was high (91.6%) and significantly associated with success (OR = 2.93; 95% CI: 1.11–7.74; *p* = 0.039). No other factors were significantly associated. **Conclusions:** While PALB remains a valid second-line therapy, its efficacy may be declining, though not statistically inferior to 80%. Improving adherence and strengthening stewardship are essential.

## 1. Introduction

*Helicobacter pylori* (*H. pylori*) colonises the gastric mucosa of an estimated 4.4 billion people worldwide, accounting for >50% of the global population and remaining a leading aetiological factor for chronic gastritis, peptic-ulcer disease, mucosa-associated lymphoid-tissue lymphoma, and non-cardia gastric cancer [1]. In Vietnam, serological and urea-breath surveys consistently report prevalence rates between 50% and 70%, among the highest in Southeast Asia [2,3]. Given the country’s high gastric cancer incidence and the direct healthcare costs of ulcer disease, effective eradication strategies are a national priority. Meta-analyses have confirmed that successful *H. pylori* eradication reduces peptic-ulcer recurrence, prevents progression of atrophic gastritis, and lowers long-term gastric cancer risk [4].

Current first-line treatment in Vietnam follows the Maastricht VI/Florence Consensus and the 2022 Vietnamese Association of Gastroenterology (VNAGE) guideline, both of which recommend a 14-day classical bismuth-based quadruple regimen (proton-pump inhibitor [PPI] + bismuth + tetracycline + metronidazole) [5,6]. However, skyrocketing antimicrobial resistance has eroded the effectiveness of this and other empirical regimens. National surveillance shows clarithromycin resistance >30%, metronidazole resistance >70%, and a worrying rise in amoxicillin (≈20%) and fluoroquinolone (≈35%) resistance over the past decade [7]. These trends mirror the World Health Organisation’s designation of clarithromycin-resistant *H. pylori* as a “high-priority” pathogen for new-drug development [8]. Globally, *H. pylori* infection affects approximately 50% of the population, with higher prevalence in Asia, Africa, and Latin America. According to a recent meta-analysis, resistance to levofloxacin has been rising steadily across Asia, with rates reaching 20–40% [1,2]. These trends pose challenges to the effectiveness of second-line regimens in various settings.

Levofloxacin-based quadruple therapy (PALB: PPI + amoxicillin + levofloxacin + bismuth) is therefore endorsed as a second-line option after failure of classical bismuth-based quadruple therapy [5]. Yet Vietnamese evidence remains sparse and dated; small single-centre studies performed before 2020 reported eradication rates ranging from 69.6% to 93.1% [9], figures obtained before the recent surge in fluoroquinolone use for respiratory and COVID-19–related infections. Consequently, clinicians lack contemporary real-world data to judge whether PALB still achieves the ≥80% intention-to-treat (ITT) benchmark recommended by international consensus.

Against this background, we conducted a prospective cohort study with the following three objectives: (i) to determine the current eradication rate of PALB in Vietnamese patients who failed first-line therapy; (ii) to quantify patient adherence to the 14-day regimen; and (iii) to identify demographic or treatment-related factors associated with eradication success. By providing up-to-date effectiveness data, our study seeks to inform national guideline updates and contribute to global discussions on optimising second-line *H. pylori* therapy in regions of escalating antibiotic resistance.

## 2. Results

### 2.1. Baseline Characteristics

A total of 225 patients met the inclusion criteria and were enrolled in the study. The mean age was 43.7 ± 13.1 years, with 84 males (37.3%) and 141 females (62.7%). Smoking history was present in 17.8% of participants. Most patients (76.0%) had previously received a bismuth-based quadruple therapy as their first-line regimen, while 15.6% had undocumented regimens (Table 1).

### 2.2. H. pylori Eradication Rates

Of the 225 patients, 206 were deemed adherent (≥80% of medication taken correctly) and were thus included in the per-protocol (PP) analysis. All enrolled patients were included in the modified intention-to-treat (mITT) analysis. The eradication rate was 78.6% in the PP analysis and 78.2% in the mITT population (Table 2).

The 95% confidence intervals for eradication rates were 72.8–83.6% (mITT) and 73.0–84.2% (PP), both of which include the 80% benchmark. Exact binomial tests yielded non-significant *p*-values (mITT *p* = 0.28; PP *p* = 0.34), indicating that the observed differences are not statistically significant. These rates are slightly below the 80% threshold recommended by current guidelines for acceptable efficacy in second-line therapy.

### 2.3. Factors Associated with Treatment Outcomes

Univariate analyses were conducted to assess associations between patient characteristics and eradication success (Table 3). No statistically significant differences were observed for age, sex, smoking status, or levofloxacin dosage. However, patients receiving higher levofloxacin doses (>500 mg/day) had numerically higher eradication rates (87.1% in PP analysis, *p* = 0.052), approaching statistical significance.

Importantly, treatment adherence was the only factor significantly associated with eradication success. Patients with good adherence had a mITT eradication rate of 80.1%, compared to 57.9% among those with poor adherence (*p* = 0.039). The calculated odds ratio for eradication success in adherent patients was 2.93 (95% CI: 1.11–7.74), indicating a nearly threefold increase in the likelihood of successful treatment.

## 3. Discussion

This prospective, real-world cohort provides the most up-to-date Vietnamese evidence on the performance of levofloxacin-based quadruple therapy (PALB) as a rescue regimen. The overall eradication rate of ~78% (both mITT and PP) is numerically below the internationally accepted 80% benchmark. However, the 95% confidence intervals include this threshold, and statistical testing showed no significant difference. This suggests a descriptive trend rather than definitive statistical inferiority. Nonetheless, the numerically lower eradication rate may signal an incremental but clinically meaningful erosion of PALB efficacy in the post-COVID-19 era.

### 3.1. Declining Efficacy in the Context of Rising Resistance

Earlier domestic series reported PP success rates of 89–93% when PALB was used as first- or second-line therapy [10,11]. The ≥ 10-percentage-point drop observed in the current cohort parallels microbiological surveillance showing a sharp increase in levofloxacin resistance from <20% in 2015 to >50% in 2022, together with a smaller but notable rise in amoxicillin resistance (>60%) [7]. These trends are plausibly linked to the surge in community fluoroquinolone use during the SARS-CoV-2 pandemic and to the widespread availability of over-the-counter antibiotics in Vietnam. Similar declines have been reported in China and Thailand, where second-line levofloxacin regimens now achieve ITT cure rates of only 70–75% [12,13].

### 3.2. Adherence: The Modifiable Driver of Success

Treatment adherence emerged as the sole independent predictor of eradication, with adherent patients being nearly three times more likely to achieve cure (aOR ≈ 3.0). This mirrors data from a large Thai registry in which good adherence conferred a 36-fold higher chance of success [14]. Importantly, PALB is intrinsically “patient-friendly” as follows: twice-daily dosing, absence of tetracycline-related gastritis, and fewer metallic-taste complaints than with metronidazole-rich regimens. These features probably explain the high adherence rate (91.6%) in our real-world setting and justify continued PALB use where antimicrobial susceptibility testing (AST) is not feasible.

### 3.3. Clinical and Public-Health Implications

*Choice of rescue regimen*—In urban Vietnamese centres with AST capability, bismuth-based quadruple therapy guided by culture or molecular testing should remain the first choice. Where AST is unavailable, empirical PALB still offers an acceptable balance between efficacy, tolerability, and cost—but clinicians should anticipate ~20% failure and arrange early retesting.

*Antibiotic stewardship*—The present findings reinforce the urgent need for national surveillance programmes and tighter regulation of outpatient antibiotic sales. We advocate for the adoption of a “test–treat–track” algorithm as follows: routine UBT confirmation, resistance-guided second-line therapy whenever possible, and centralised reporting of eradication failures to inform annual guideline updates.

*Patient-centred counselling*—Because even modest improvements in adherence translate into substantial gains in cure probability, clinicians should allocate time to clarify dosing schedules, reinforce the importance of completing the 14-day course, and proactively manage adverse events.

### 3.4. Study Strengths and Limitations

Strengths include prospective design, stringent follow-up (0% loss), and reporting in line with the *H. pylori* Treatment-Outcome Checklist. Limitations include our single-centre scope, lack of resistance testing, and the sample size being insufficient to confirm the borderline benefit of high-dose levofloxacin. Although higher levofloxacin doses (>500 mg/day) were associated with numerically greater eradication rates (87.1%), this difference did not reach statistical significance (*p* = 0.052). Thus, the observed trend may indicate potential dose–response effects, but further research is needed. Multicentre trials incorporating genotypic fluoroquinolone-resistance testing (e.g., *gyrA* mutations) are warranted.

## 4. Methods

### 4.1. Study Design and Setting

This was a prospective, real-world cohort study conducted at the Department of Gastroenterology Clinic, University Medical Center Ho Chi Minh City, Vietnam. The study period spanned from January to December 2024. The protocol followed STROBE recommendations for observational studies and the latest *H. pylori* treatment-outcome reporting checklist proposed by Malfertheiner et al. [5].

### 4.2. Participants

Adults (≥18 years) with documented failure of first-line eradication therapy were screened consecutively during routine follow-up visits. Treatment failure was defined as either (i) a positive ^13^C-urea breath test (UBT) or (ii) a positive rapid-urease test (RUT) obtained ≥4 weeks after completion of the initial regimen, in line with Maastricht VI criteria [5].

### 4.3. Inclusion Criteria

Age ≥ 18 years;

Confirmed first-line failure;

Ability and willingness to provide informed consent.

### 4.4. Exclusion Criteria

Decompensated cirrhosis, stage 4–5 chronic kidney disease, NYHA class III–IV heart failure;

Pregnancy or breastfeeding;

Known allergy/intolerance to proton-pump inhibitors (PPIs), amoxicillin, levofloxacin or bismuth;

Use of systemic antibiotics or bismuth compounds within 4 weeks, or PPIs/H_2_-antagonists within 2 weeks, prior to enrolment;

Loss to follow-up.

### 4.5. Intervention

All eligible patients received a 14-day course of levofloxacin-based quadruple therapy consisting of the following:

PPI (standard dose, twice daily);

Amoxicillin 1000 mg, twice daily;

Levofloxacin 500–1000 mg/day;

Bismuth subcitrate 120 mg, two tablets twice daily.

Patients were instructed to abstain from taking any antibiotics or bismuth-containing compounds within 4 weeks, and acid-suppressive therapy (PPI or H2 blockers) within 2 weeks prior to the follow-up test.

### 4.6. Outcomes

The primary endpoint was eradication of *H. pylori*, assessed 4–12 weeks post-therapy by UBT and/or RUT. The secondary endpoints were (i) patient adherence and (ii) factors associated with eradication success. Adherence was calculated from pill counts and patient diaries; intake of ≥80% of prescribed doses defined good adherence [15].

### 4.7. Flow of Participants

**Figure 1:**
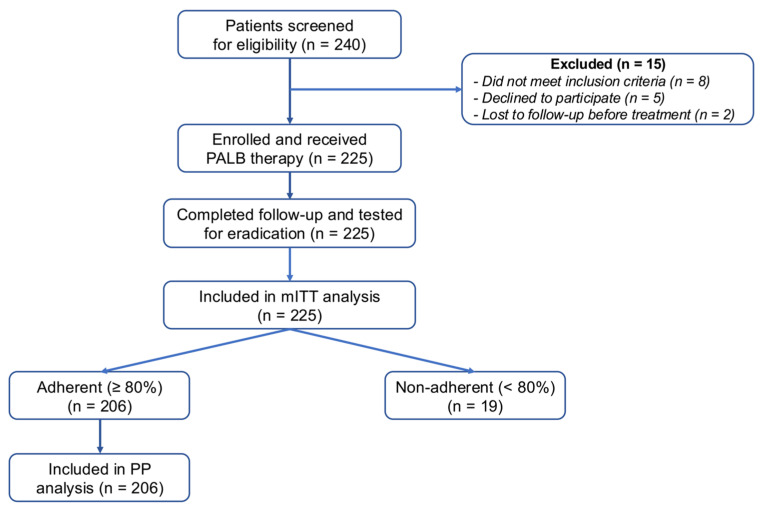
Flowchart of participant enrolment, exclusion, treatment with PALB, and assessment of *H. pylori* eradication outcome.

Figure 1 (study CONSORT-style flow diagram) illustrates the screening, exclusions, treatment allocation, and analysis sets (modified intention-to-treat [mITT] and per-protocol [PP]).

### 4.8. Statistical Analysis

Data were analysed with SPSS v26.0 (IBM, Armonk, NY, USA). Continuous variables are presented as the mean ± SD or median (IQR) and categorical variables as *n* (%). Eradication rates are reported for the following:

mITT—all participants with outcome assessment, regardless of adherence;

PP—only participants with good adherence and no major protocol deviations.

Comparisons between categorical variables employed Pearson’s χ^2^ or Fisher’s exact test; continuous variables used Student’s *t*-test or Mann–Whitney *U* where appropriate. Variables with *p* < 0.20 in univariate analysis entered a multivariate logistic-regression model to identify independent predictors of eradication; adjusted odds ratios (aOR) with 95% confidence intervals (CI) were calculated. A two-sided *p* < 0.05 denoted statistical significance.

## 5. Conclusions

In this prospective real-world study, levofloxacin-based quadruple therapy (PALB) demonstrated moderate and clinically relevant efficacy as a second-line regimen for *Helicobacter pylori* eradication in Vietnam, with eradication rates of approximately 78% by both mITT and PP analyses. Although this falls marginally below the internationally accepted 80% benchmark, the regimen remains a rational empirical choice in resource-constrained settings where AST is unavailable.

The observed decline in efficacy compared to earlier Vietnamese studies likely reflects escalating fluoroquinolone and amoxicillin resistance, a consequence of widespread antibiotic use and limited stewardship. Crucially, treatment adherence emerged as the strongest independent predictor of success, underscoring the importance of structured patient counselling and support.

These findings provide timely, context-specific data that inform national treatment guidelines and highlight the urgent need for updated resistance surveillance, stewardship frameworks, and policies regulating antibiotic access. As Vietnam continues to face high *H. pylori* prevalence and gastric cancer burden, maintaining the effectiveness of existing regimens through patient-centred care and antibiotic policy reform will be essential.

## Figures and Tables

**Table 1:** First-line *H. pylori* eradication regimens used prior to study enrolment.

First-Line Regimen	No. of Patients (*n*)	Percentage (%)
***Classical bismuth-based quadruple therapy (PPI + AMX + MET + BIS)***	171	76.0
***PPI + MET + CLR + BIS***	6	2.7
***PPI + MET + AMX + BIS***	4	1.8
***PPI + AMX + CLR***	4	1.8
***PPI + MET + AMX***	2	0.9
***PPI + AMX + CLR + BIS***	2	0.9
***PPI + MET + AMX + CLR***	1	0.4
***Unspecified regimen***	35	15.6

PPI—Proton Pump Inhibitor; AMX—Amoxicillin; MET—Metronidazole; CLR—Clarithromycin; BIS—Bismuth Subcitrate.

**Table 2:** *H. pylori* eradication rates by analysis type.

Analysis Type	No. of Patients with Eradication (*n*/N)	Eradication Rate (%)	95% CI
***Modified Intention-to-Treat (mITT)***	176/225	78.2	72.8–83.6
***Per-Protocol (PP)***	162/206	78.6	73.0–84.2

Note: The eradication rate by mITT includes all patients who returned for follow-up, regardless of adherence. The PP analysis includes only those with ≥80% adherence. Both analyses indicate modest efficacy below the 80% benchmark recommended by international guidelines.

**Table 3:** *H. pylori* eradication rates by patient characteristics.

Patient Characteristic	Eradication Rate (mITT) *n*/N (%)	Eradication Rate (PP) *n*/N (%)	*p*-Value
***Age***			
***<60 years***	153/194 (78.9%)	141/178 (79.2%)	0.558
***≥60 years***	23/31 (74.2%)	21/28 (75.0%)	0.623
***Male***	65/84 (77.4%)	59/75 (78.7%)	0.813
***Female***	111/141 (78.7%)	103/131 (78.6%)	0.995
***Smoker***	31/40 (77.5%)	29/38 (76.3%)	0.903
***Non-smoker***	145/185 (78.4%)	133/168 (79.2%)	0.699
***Levofloxacin 500 mg/day***	120/160 (75.0%)	108/144 (75.0%)	0.066
***Levofloxacin > 500 mg/day***	56/65 (86.2%)	54/62 (87.1%)	0.052
***Good adherence (≥80%)***	165/206 (80.1%)	165/206 (80.1%)	0.039 *
***Poor adherence (<80%)***	11/19 (57.9%)	-	-

Note: Eradication rates are shown for both modified intention-to-treat (mITT) and per-protocol (PP) analyses. Only treatment adherence demonstrated a statistically significant association with eradication success (*p* < 0.05). Other factors, including age, sex, smoking status, and levofloxacin dosage, showed no significant impact. * *p* < 0.05 indicates statistical significance.

## Data Availability

The datasets used in this study are available upon request from the corresponding authors.

## Abbreviations

The following abbreviations are used in this manuscript:

*H. pylori*	*Helicobacter pylori*
PALB	Levofloxacin-based quadruple therapy (proton pump inhibitor + amoxicillin + levofloxacin + bismuth)
PPI	Proton pump inhibitor
AMX	Amoxicillin
MET	Metronidazole
CLR	Clarithromycin
BIS	Bismuth subcitrate
mITT	Modified intention-to-treat
PP	Per-protocol
UBT	Urea breath test
RUT	Rapid urease test
AST	Antimicrobial susceptibility testing
OR	Odds ratio
CI	Confidence interval
SD	Standard deviation
IQR	Interquartile range
